# Inhibition of XPO1 enhances cell death induced by ABT‐199 in acute myeloid leukaemia via Mcl‐1

**DOI:** 10.1111/jcmm.13886

**Published:** 2018-09-14

**Authors:** Daniel A. Luedtke, Yongwei Su, Shuang Liu, Holly Edwards, Yue Wang, Hai Lin, Jeffrey W. Taub, Yubin Ge

**Affiliations:** ^1^ Cancer Biology Graduate Program Wayne State University School of Medicine Detroit MI USA; ^2^ National Engineering Laboratory for AIDS Vaccine School of Life Sciences Jilin University Changchun China; ^3^ Department of Pediatrics Wayne State University School of Medicine Detroit MI USA; ^4^ Department of Oncology Wayne State University School of Medicine Detroit MI USA; ^5^ Molecular Therapeutics Program Karmanos Cancer Institute Wayne State University School of Medicine Detroit MI USA; ^6^ Department of Pediatric Hematology and Oncology The First Hospital of Jilin University Changchun China; ^7^ Department of Hematology and Oncology The First Hospital of Jilin University Changchun China; ^8^ Division of Pediatric Hematology and Oncology Children's Hospital of Michigan Detroit MI USA

**Keywords:** ABT‐199, acute myeloid leukaemia, Bcl‐2, KPT‐330, XPO1

## Abstract

The antiapoptotic Bcl‐2 family proteins play critical roles in resistance to chemotherapy in acute myeloid leukaemia (AML). The Bcl‐2‐selective inhibitor ABT‐199 (Venetoclax) shows promising antileukaemic activity against AML, though Mcl‐1 limits its antileukaemic activity. XPO1 is a nuclear exporter overexpressed in AML cells and its inhibition decreases Mcl‐1 levels in cancer cells. Thus, we hypothesized that the XPO1‐selective inhibitor KPT‐330 (Selinexor) can synergize with ABT‐199 to induce apoptosis in AML cells through down‐regulation of Mcl‐1. The combination of KPT‐330 and ABT‐199 was found to synergistically induce apoptosis in AML cell lines and primary patient samples and cooperatively inhibit colony formation capacity of primary AML cells. KPT‐330 treatment decreased Mcl‐1 protein after apoptosis initiation. However, binding of Bim to Mcl‐1 induced by ABT‐199 was abrogated by KPT‐330 at the same time as apoptosis initiation. KPT‐330 treatment increased binding of Bcl‐2 to Bim but was overcome by ABT‐199 treatment, demonstrating that KPT‐330 and ABT‐199 reciprocally overcome apoptosis resistance. Mcl‐1 knockdown and overexpression confirmed its critical role in the antileukaemic activity of the combination. In summary, KPT‐330 treatment, alone and in combination with ABT‐199, modulates Mcl‐1, which plays an important role in the antileukaemic activity of the combination.

## 1 INTRODUCTION

Despite substantially enhanced understanding of the biology of acute myeloid leukaemia (AML), standard chemotherapy (cytarabine plus an anthracycline, e.g. daunorubicin) and bone marrow transplant have remained the main treatment option for patients with AML over the course of the last four decades. This lack of change in standard of care has led to a meagre improvement in AML survival compared to other leukaemia types. Although many fit patients respond initially to standard therapy, survival rates remain low because of disease relapse. Leukaemic stem cells (LSCs), and our inability to target them, are believed to be responsible for AML relapse.^1^

Bcl‐2 is overexpressed in bulk AML cells and LSCs, making it a promising therapeutic target for the treatment of AML.^2^ Anti‐apoptotic Bcl‐2 family proteins (e.g., Bcl‐2, Bcl‐xL, and Mcl‐1) sequester pro‐apoptotic Bcl‐2 family proteins, such as Bim, to prevent induction of apoptosis.^3^ The Bcl‐2 family has been found to be dysregulated in AML, with increases in Bcl‐2, Bcl‐xL, and/or Mcl‐1 expression associated with chemotherapy resistance and poor prognoses.^2^, ^4^, ^5^, ^6^, ^7^ Mcl‐1 was previously shown to be necessary to inhibit Bak and Bax activation.^8^, ^9^, ^10^ Thus, targeting the anti‐apoptotic Bcl‐2 family proteins represent a promising strategy for the treatment of AML. Although inhibitors of this family have been promising, inhibition of Bcl‐xL has been associated with platelet death and subsequent thrombocytopenia,^5^ which has sparked interest in the Bcl‐2‐selective inhibitor ABT‐199 (Venetoclax). Its excellent antileukaemic activity against chronic lymphocytic leukaemia led to FDA approval in April 2016. Our group and others have reported that ABT‐199 has promising activity against preclinical models of AML and other cancers.^11^, ^12^, ^13^, ^14^, ^15^, ^16^, ^17^ Results of a phase II clinical trial have shown that ABT‐199 has promising clinical activity compared to current alternatives, but relapse remains a concern.^18^, ^19^ Combinations such as ABT‐199 with decitabine or azacitidine can help induce remission but lack improvement in overall survival.^20^ Thus, new therapeutic combinations involving ABT‐199 are urgently needed to eradicate bulk AML cells and LSCs responsible for relapse.

Bcl‐2 sequesters Bim, preventing Bim from inducing apoptosis. We have shown that the small molecule inhibitor ABT‐199 can disrupt this interaction in AML.^21^ However, ABT‐199 treatment also caused increased sequestration of Bim by Mcl‐1 in ABT‐199‐resistant AML cells.^21^ Previous studies by our lab and others have shown that ABT‐199 treatment increases levels of Mcl‐1 in resistant AML cells lines and primary AML patient samples by enhancing Mcl‐1 protein stability rather than gene transcription.^14^, ^22^ Furthermore, CRISPR knockdown of Mcl‐1 significantly enhanced ABT‐199‐induced apoptosis in AML cells. These results demonstrate that Mcl‐1 is a key player in the intrinsic resistance to ABT‐199 in AML cells. Thus, targeting Mcl‐1 either directly or indirectly represents a promising means to overcome ABT‐199 resistance in AML.

We previously demonstrated that the Mcl‐1 small molecule inhibitor A‐1210477 was able to synergize with ABT‐199 to induce apoptosis in AML cell lines and primary patient samples by disrupting the Bim‐Mcl‐1 interaction.^23^ However, the drug combination also synergized to reduce proliferation of normal peripheral blood mononuclear cells (PMNCs), albeit at higher concentrations. Thus, indirect inhibition of Mcl‐1 may prove to be useful in combination with ABT‐199.

Exportin 1 (XPO1), also known as chromosome region maintenance 1, is a protein which regulates the nuclear export of client proteins and has been found to play a critical role in many cancers including prostate cancer, ovarian cancer, osteosarcoma, glioma, cervical cancer, chronic lymphoid leukaemia (CLL), non‐Hodgkin's lymphoma, multiple myeloma, and AML.^24^, ^25^, ^26^, ^27^, ^28^ As a nuclear exporter, XPO1 plays a critical role in the intracellular localization of many proteins, as well as some mRNA transcripts, and is required for survival of solid tumors and hematological malignancies.^26^, ^29^, ^30^, ^31^, ^32^, ^33^, ^34^ KPT‐330 (Selinexor) is a XPO1‐selective inhibitor that is currently being tested in phase I and phase II clinical trials for hematological malignancies and solid tumors (NCT02091245, NCT02530476, NCT02249091, NCT02419495, NCT02343042, and NCT02178436). Preclinical results show that KPT‐330 induces responses at well‐tolerated doses 35 and decreases Mcl‐1 protein levels.^29^ Higher XPO1 levels are associated with poor prognosis and targeting XPO1 by KPT‐330 has shown promise in clinical trials in targeting AML.^26^, ^32^ Our working hypothesis was that because XPO1 inhibition decreases Mcl‐1 protein levels, targeting XPO1 can synergize with ABT‐199 treatment in AML.

## 2 MATERIALS AND METHODS

### 2.1 Drugs

ABT‐199, KPT‐330, and KPT‐8602 were purchased from Selleck Chemicals. Z‐VAD‐FMK was purchased from BD Biosciences (Franklin Lakes, NJ, USA). Cycloheximide was purchased from Sigma‐Aldrich (St Louis, MO, USA).

### 2.2 Cell culture

MV4‐11 and THP‐1 cell lines were purchased from the American Type Culture Collection (Manassas, VA, USA). CTS was a gift from Dr. A Fuse from the National Institute of Infectious Diseases (Tokyo, Japan). OCI‐AML3 was purchased from the German Collection of Microorganisms and Cell Cultures (DSMZ). MOLM‐13 was purchased from AddexBio (San Diego, CA, USA). The cell lines were cultured as previously described,^36^ and were authenticated in August 2017 at the Genomics Core at Karmanos Cancer Institute using the PowerPlex^®^ 16 System from Promega (Madison, WI, USA). The cell lines were tested for the presence of mycoplasma by PCR on a monthly basis.^37^

Diagnostic AML patient samples and peripheral blood mononuclear cells (PMNCs) from healthy donors were purified by standard Ficoll‐Hypaque density centrifugation, then cultured in RPMI 1640 (ThermoFisher, Waltham, MA, USA) with 20% foetal bovine serum (ThermoFisher), ITS Solution (Sigma‐Aldrich), and 20% supernatant of the 5637 bladder cancer cell line (as a source of granulocyte‐macrophage colony‐stimulating factor, granulocyte colony stimulating factor, interleukin‐1 beta, macrophage colony‐stimulating factor, and stem cell factor^14^, ^38^, ^39^).

### 2.3 Clinical samples

Diagnostic AML patient samples were obtained from the First Hospital of Jilin University (Table 1). Written informed consent was provided according to the Declaration of Helsinki. This study was approved by the Human Ethics Committee of The First Hospital of Jilin University. Clinical samples were screened for gene mutations and fusion genes as described previously.^14^, ^40^

**Table 1:** Patient characteristics for primary AML patient samples

Patient	Gender	Age (year)	Disease status	FAB subtype	Cytogenetics	Blast purity (%)	Gene mutation
AML#114	Female	60	Relapsed	M2	46, XX	69.0	CEBPα double mutation
AML#115	Male	40	Newly diagnosed	M2	46, XY	88.5	CEBPα double mutation
AML#116	Female	51	Relapsed	M5	46, XX	94.0	Flt‐3 ITD, NPM1, DNMT3A
AML#121	Male	49	Relapsed	M2	46, XY	79.5	Flt‐3 ITD, NPM1
AML#122	Male	45	Newly diagnosed	M2	47, XY, −8, +11, +14	78.0	Dup MLL
AML#123	Male	24	Newly diagnosed	M5	46, XY	92.0	CNSL
AML#124	Male	19	Newly diagnosed	M4	46, XY	62.5	CEBPA double mutation, c‐Kitv, NRAS, GATA2, Flt‐3 S451F
AML#126	Female	60	Relapsed	M2	46, XX	49.0	CEBPA double mutation
AML#127	Male	77	Newly diagnosed	M4	NA	NA	NA
AML#128	Male	66	Newly diagnosed	M5	46, XY	41.0	ND
AML#130	Male	66	Newly diagnosed	M2	46, XY	NA	CEBPA double mutation
AML#131	Female	40	Newly diagnosed	M4	46, XX	53.5	NPM‐1, IDH‐1
AML#132	Female	48	Newly diagnosed	M2	NA	61.0	NA

NA: not available; ND: not detected.

### 2.4 Western blot analysis

Cells were lysed in the presence of protease and phosphatase inhibitors (Roche Diagnostics, Indianapolis, IN, USA). Whole cell lysates were subjected to SDS‐polyacrylamide gel electrophoresis, electrophoretically transferred onto polyvinylidene difluoride membranes (Thermo Fisher Scientific, Waltham, MA, USA) and immunoblotted with anti‐Bcl‐2 (Abcam, Cambridge, MA, USA), ‐Bcl‐xL, ‐Mcl‐1, ‐PARP, ‐Bim, ‐Bak, ‐Bax, ‐cleaved caspase‐3 (designated ‐cf‐Cas3; Cell Signaling Technology, Danvers, MA, USA), or ‐β‐actin (Sigma‐Aldrich) antibody, as previously described.^41^, ^42^ Immunoreactive proteins were visualized using the Odyssey Infrared Imaging System (Li‐Cor, Lincoln, NE, USA), as described by the manufacturer. Western blots were repeated at least 2 times and one representative blot is shown. Densitometry measurements were made using Odyssey V3.0 (Li‐Cor), normalized to β‐actin, and calculated as the fold change compared to the corresponding no drug treatment control.

### 2.5 Annexin V/propidium iodide staining and flow cytometry analysis

Acute myeloid leukaemia cells were treated with ABT‐199 or KPT‐330, alone or in combination, and subjected to flow cytometry analysis using the annexin V‐fluorescein isothiocyanate (FITC)/propidium iodide (PI) Apoptosis Kit (Beckman Coulter, Brea, CA, USA), as previously described.^43^, ^44^ Results are expressed as percent annexin V+ cells. For the AML cell lines, experiments were performed 3 independent times in triplicate and data presented are from one representative experiment, while primary patient sample experiments were performed once in triplicate due to limited sample. Patient samples were chosen based on availability of adequate sample for the assay. The extent and direction of antileukaemic interaction was determined by calculating the combination index (CI) values using CompuSyn software (Combosyn Inc., Paramus, NJ, USA). CI <1, CI = 1, and CI >1 indicate synergistic, additive, and antagonistic effects, respectively.^43^, ^45^

### 2.6 shRNA knockdown and pLOC overexpression

The pMD‐VSV‐G and delta 8.2 plasmids were gifts from Dr. Dong at Tulane University. Bim and non‐target control (NTC) shRNA lentiviral vectors were purchased from Sigma‐Aldrich. Precision LentiORF Mcl‐1 and RFP (red fluorescent protein) lentivirus vector were purchased from Dharmacon (Lafayette, CO, USA). Lentivirus production and transduction were carried out as previously described.^46^ Briefly, TLA‐HEK293T cells were transfected with pMD‐VSV‐G, delta 8.2, and lentiviral shRNA or Precision LentiORF constructs using Lipofectamine and Plus reagents (Life Technologies, Carlsbad, CA, USA) according to the manufacturer's instructions. Virus containing culture medium was harvested 48 hours post transfection. Cells were transduced overnight using 1 mL of virus supernatant and 4 μg of polybrene and then cultured for an additional 48 hours prior to selection with puromycin or blasticidin.

### 2.7 CRISPR knockdown

The lentiCRISPRv2 plasmid was a gift from Feng Zhang at the Broad Institute of MIT and Harvard (Addgene plasmid #52961). Guide RNAs were designed using the CRISPR design tool (http://crispr.mit.edu). The NTC and Mcl‐1 vectors were generated using Feng Zhang's protocol, which is available on Addgene's website (http://www.addgene.org). Lentivirus production and transduction were carried out as described above in “shRNA Knockdown,” except that psPAX2 (gift from Didier Trono at the Swiss Institute of Technology, Addgene plasmid #12260) was used instead of delta 8.2.

### 2.8 Colony forming assay

Primary AML patient samples were treated with either ABT‐199 or KPT‐330, alone or in combination, for 24 hours. The cells were washed three times with PBS and then plated in MethoCult (Stem Cell Technologies, Vancouver, Canada) and allowed to incubate at 37°C humidified atmosphere containing 5% CO_2_/95% air for 14‐16 days. Colony forming units (CFUs) were visualized using an inverted microscope and the number of colonies containing >50 cells were counted. Technical triplicates were performed.

### 2.9 RT‐PCR

Total RNA was extracted using TRIzol (Thermo Fisher Scientific) and cDNAs were prepared from 2 μg total RNA using random hexamer primers and a RT‐PCR Kit (Thermo Fisher Scientific), and then purified using the QIAquick PCR Purification Kit (Qiagen, Germantown, MD, USA), as described previously.^44^ Mcl‐1 (cat no. Hx01050896_m1) and GAPDH transcripts were quantitated using TaqMan probes (Thermo Fisher Scientific) and a LightCycler 480 real‐time PCR machine (Roche Diagnostics), based on the manufacturer's instructions. Real‐time PCR results were expressed as means from three independent experiments and were normalized to GAPDH transcripts. Fold changes were calculated using the comparative Ct method.^47^

### 2.10 MTT assays

MTT (3‐[4,5‐dimethyl‐thiazol‐2‐yl]‐2,5‐diphenyltetrazoliumbromide; Sigma‐Aldrich) assays were performed as previously described.^36^, ^43^, ^48^ Human PMNC samples were treated with variable concentrations of ABT‐199 and/or KPT‐330 for 72 hours. For each PMNC sample, the MTT assay was performed once due to limited sample.

### 2.11 Statistical analysis

Differences in cell death and colony formation between treated (individually or combined) and untreated cells or combined and individual drug treatment were compared using the pairwise two‐sample *t* test or repeated measures one‐way ANOVA with Bonferroni post hoc test. Statistical analyses were performed with GraphPad Prism 5.0 (GraphPad Software, LaJolla, CA, USA). Error bars represent ±SEM. The level of significance was set at *P* < 0.05.

## 3 RESULTS

### 3.1 Inhibition of XPO1 synergizes with ABT‐199 in AML cell lines

**Figure 1:**
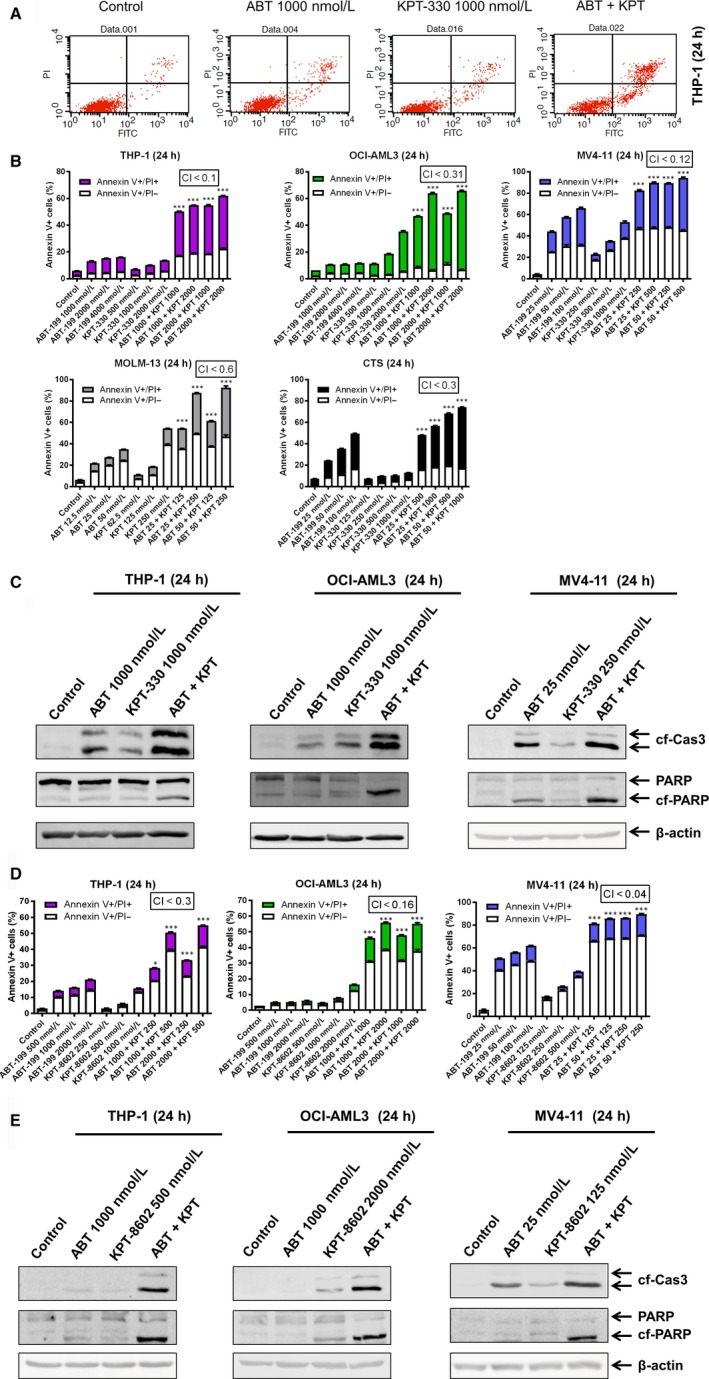
Inhibition of XPO1 synergizes with ABT‐199 in AML cell lines. (A, B, D) Annexin V‐FITC/PI staining and flow cytometry analyses were performed following 24 hours treatment with ABT‐199 and/or XPO1 inhibitor KPT‐330 or KPT‐8602. (A) Representative dot plots for THP‐1 cells. (B and D) The results are graphed as mean percent of annexin V+ cells ± SEM, ***P < 0.001. Combination index (CI) values were calculated using CompuSyn software (B and D). (C and E) Whole cell lysates from THP‐1, OCI‐AML3, and MV4‐11 cells treated with ABT‐199 or KPT‐330/KPT‐8602, alone or in combination, for 24 hours, were subjected to Western blotting and probed with the indicated antibodies

To begin to test our hypothesis that KPT‐330 can synergize with ABT‐199 to induce apoptosis, we tested various concentrations of ABT‐199 and KPT‐330, alone and in combination, in five AML cell lines. The CI was used to determine synergy.^45^ At 24 hours, synergy was observed between the two drugs in THP‐1 (CI <0.1), OCI‐AML3 (CI <0.31), MV4‐11 (CI <0.12), MOLM‐13 (CI <0.6), and CTS (CI <0.3) cell lines (Figure 1A and B). Cleavage of PARP and caspase 3 were strongly enhanced in the combination treatment when compared to ABT‐199 or KPT‐330 alone in THP‐1, OCI‐AML3, and MV4‐11 cells (Figure 1C) and this synergy was found to be at least partially caspase dependent (data not shown). To further confirm our results, we used a second generation XPO1 inhibitor and KPT‐330 analogue, KPT‐8602. At 24 hours, synergy was observed between the two drugs in THP‐1 (CI <0.3), OCI‐AML3 (CI <0.16) and MV4‐11 (CI <0.04) cell lines (Figure 1D). Consistent with KPT‐330, cleavage of PARP and caspase 3 was strongly enhanced in the combination treatment when compared to ABT‐199 or KPT‐8602 alone in these AML cell lines (Figure 1E). These results show that XPO1 inhibition synergizes with ABT‐199 to induce apoptosis in AML cell lines.

### 3.2 KPT‐330 down‐regulates Mcl‐1 and disrupts its interaction with Bim

**Figure 2:**
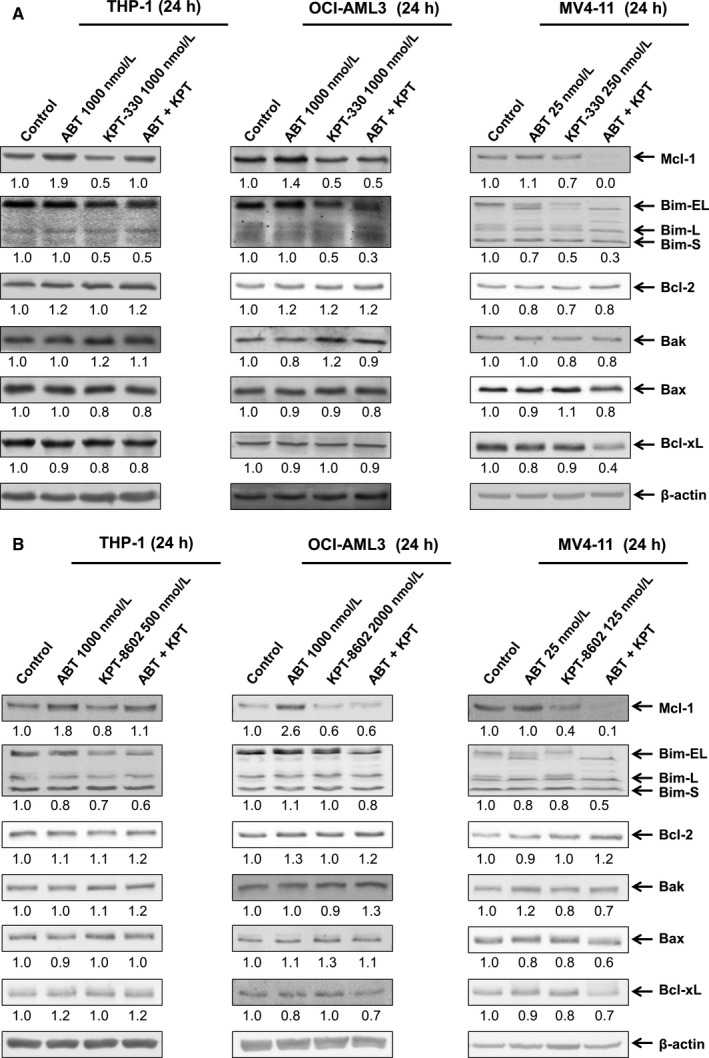
Inhibition of XPO1 down‐regulates Mcl‐1 and prevent up‐regulation of Mcl‐1 induced by ABT‐199. (A and B) THP‐1, OCI‐AML3, and MV4‐11 cells were treated with ABT‐199 and/or KPT‐330/KPT‐8602 for 24 hours. Whole cell lysates were subjected to Western blotting and probed with the indicated antibodies. Relative densitometry measurements of Mcl‐1, Bim, Bcl‐2, Bax, Bak, and Bcl‐xL were performed using Odyssey Software V3.0

Having observed the synergy between ABT‐199 and KPT‐330, we sought out to determine how the combination treatment affected levels of relevant Bcl‐2 family proteins. In agreement with our previous studies, Mcl‐1 levels increased in response to ABT‐199 treatment in the ABT‐199‐resistant cell lines (THP‐1 and OCI‐AML3), but not in the ABT‐199‐sensitive cell line MV4‐11 (Figure 2A).^21^, ^23^ In support of our hypothesis, KPT‐330 treatment decreased Mcl‐1 levels and was able to prevent up‐regulation of Mcl‐1 induced by ABT‐199. In contrast, the levels of Bcl‐2, Bak, Bax, and Bcl‐xL remained relatively unchanged. Curiously, KPT‐330 treatment alone or in combination with ABT‐199 decreased levels of Bim, which would be expected to oppose apoptosis. However, based on the previous figure, the overall effect is the induction of apoptosis. Thus, the effects of Mcl‐1 down‐regulation induced by KPT‐330 likely predominate. KPT‐8602 had similar effects as KPT‐330 on Mcl‐1 levels alone and in combination with ABT‐199 (Figure 2B). In contrast, KPT‐8602 by itself did not substantially decrease Bim protein levels. As KPT‐330 is further advanced in clinical trials, KPT‐330 was used in the rest of our study.

**Figure 3:**
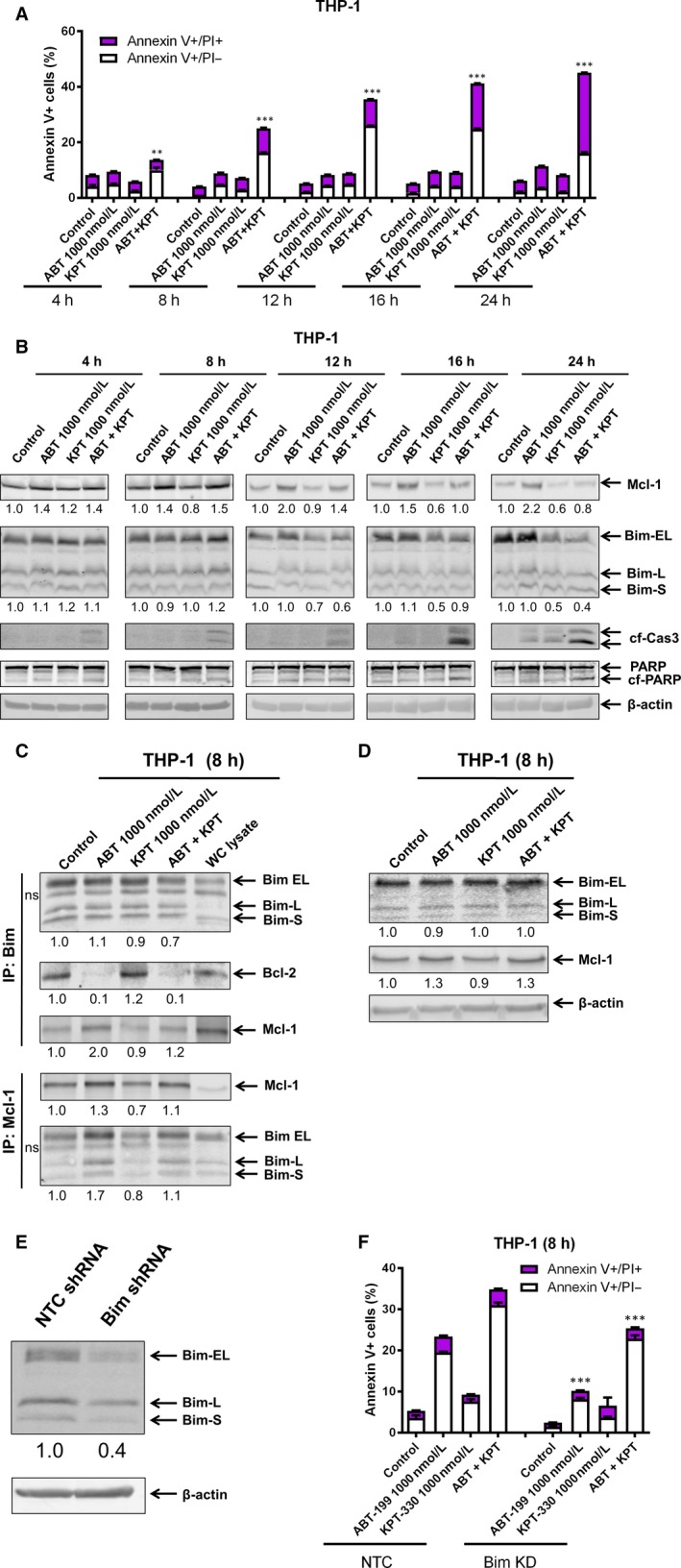
KPT‐330 disrupts the interaction between Bim and Mcl‐1. (A) THP‐1 cells were treated for up to 24 hours with ABT‐199 and KPT‐330, alone or in combination, and then subjected to annexin V/PI staining and flow cytometry analyses; **P < 0.01 and ***P < 0.001. (B) THP‐1 cells were treated with ABT‐199 and/or KPT‐330 for 0‐24 hours. Western blotting results are shown (n = 3). (C and D) THP‐1 cells were treated with ABT‐199 and/or KPT‐330 for 8 hours. Bim (top) or Mcl‐1 (bottom) were immunoprecipitated from whole cell lysates and then subjected to Western blotting and probed with the indicated antibodies (n = 2). Relative densitometry measurements of Mcl‐1, Bim, and Bcl‐2 were measured using Odyssey Software V3.0. ns indicates a nonspecific band. Accompanying inputs for the immunoprecipitation experiment are show in (D). WC indicates whole cell lysates. (E and F) shRNA knockdown of Bim and non‐template control (NTC) were generated in THP‐1 cells. Knockdown was confirmed by Western blotting (n = 3). (E) Relative densitometry measurements of Bim was measured using Odyssey Software V3.0. shRNA knockdown cells were treated for 24 hours with ABT‐199 and/or KPT‐330 (F). Annexin V/PI staining and flow cytometry analyses results are shown as means ± SEM, ***P < 0.001 (F)

To further understand the role of Bcl‐2 family proteins in the apoptotic response to ABT‐199 and KPT‐330 treatment, we ran a timecourse experiment. Annexin V/PI staining and flow cytometry analyses revealed that apoptosis initiation started around 4‐8 hours for the combination treatment in THP‐1 cells (Figure 3A). Curiously, at 8 hours, Mcl‐1 levels were largely unchanged in response to KPT‐330 treatment (Figure 3B). However, immunoprecipitation of Bim showed that at 8 hours KPT‐330 prevented increased Mcl‐1 binding to Bim in response to ABT‐199 treatment (Figure 3C, top). This was further confirmed by reciprocal Mcl‐1 immunoprecipitation (Figure 3C, bottom). Whole cell lysate inputs indicate that Bim levels remained unchanged post drug treatment (Figure 3D). Further, Mcl‐1 levels were increased in both ABT‐199 treatment alone and in combination with KPT‐330. This shows that before protein levels of Mcl‐1 and Bim change, their interaction is disrupted by combined KPT‐330 and ABT‐199 treatment. Bim levels were decreased by the 12 hours time‐point. While Bim protein level decrease occurred after apoptosis initiation, knockdown of Bim partially rescued the THP‐1 cells from apoptosis, confirming the role of Bim in response to ABT‐199 and KPT‐330 treatment (Figure 3E and F).

**Figure 4:**
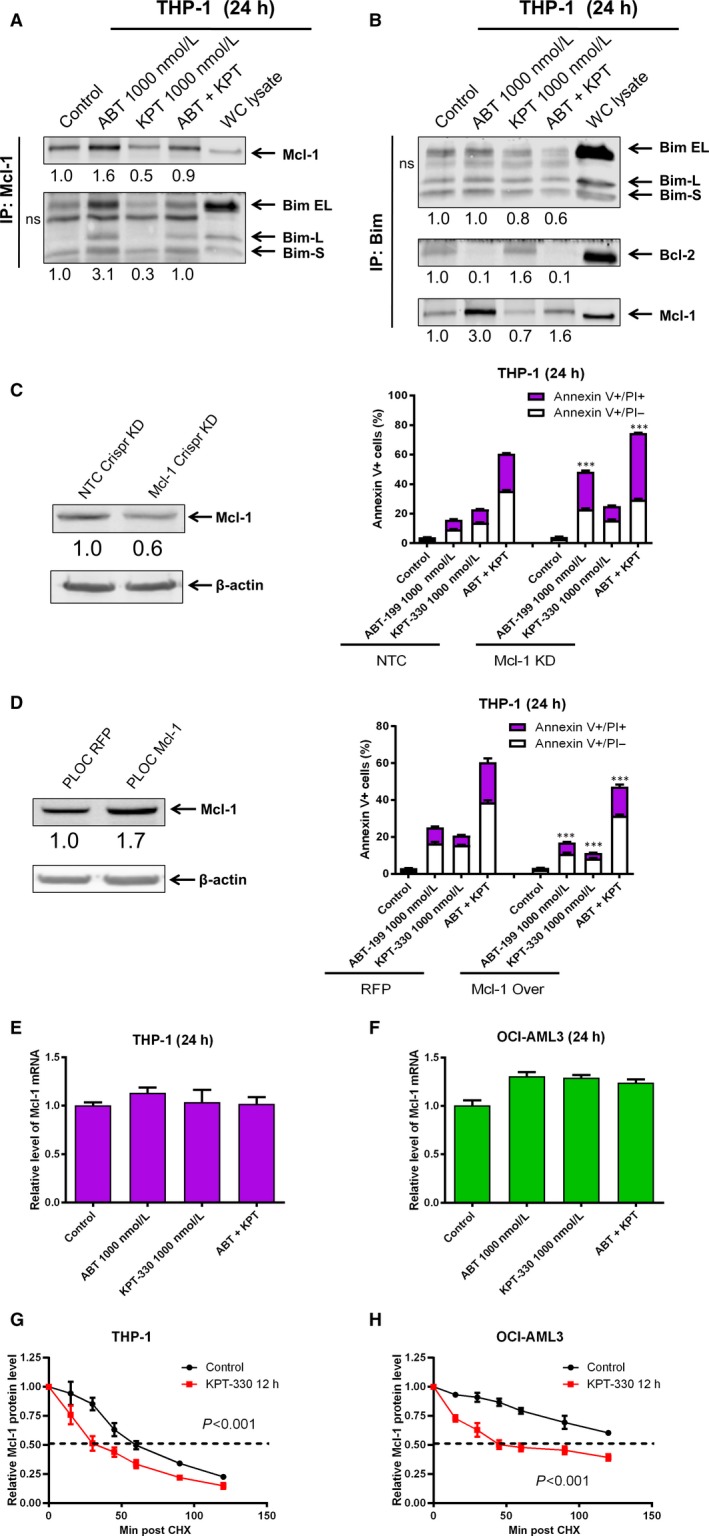
Mcl‐1 plays an important role in response to KPT‐330 and ABT‐199 treatment. (A and B) THP‐1 cells were treated with ABT‐199 and/or KPT‐330 for 24 hours. Mcl‐1 (A) and Bim (B) were immunoprecipitated from whole cell lysates and then subjected to Western blotting and probed with the indicated antibodies (n = 2). Relative densitometry measurements of Mcl‐1, Bim, and Bcl‐2 were measured using Odyssey Software V3.0. ns indicates a non‐specific band. (C and D) CRISPR knockdown and PLOC overexpression of Mcl‐1 were generated with indicated controls (NTC and RFP, respectively). Western blotting was performed to confirm knockdown or overexpression (n = 3). The cells were treated with ABT‐199 and KPT‐330, alone or in combination, for 24 hours and then subjected to annexin V/PI staining and flow cytometry analyses; ***P < 0.001. (E and F) THP‐1 and OCI‐AML3 cells were treated with ABT‐199 and/or KPT‐330 for 24 hours. RNA was extracted with TRIzol and subjected to qRT‐PCR analysis. (G and H) THP‐1 and OCI‐AML3 cells were treated with or without KPT‐330 for 12 hours followed by CHX treatment (10 μg/mL) and collected at the indicated times. Whole cell lysates were subjected to Western blotting and probed with Mcl‐1 and actin antibodies

Consistent with the 8 hours treatment and previous studies from our lab, ABT‐199 treatment for 24 hours substantially increased binding of Bim to Mcl‐1, which was prevented by the addition of KPT‐330 (Figure 4A). However, at this time‐point down‐regulation of Bim protein levels may have contributed. These results were confirmed by reciprocal immunoprecipitation with a Bim antibody (Figure 4B). Interestingly, KPT‐330 treatment for 24 hours resulted in increased binding of Bim to Bcl‐2, which was abolished by combination with ABT‐199. These results suggest that ABT‐199 and KPT‐330 reciprocally overcome resistance to apoptosis. To further determine the role of Mcl‐1 in ABT‐199‐ and KPT‐330‐induced apoptosis, a CRISPR knockdown of Mcl‐1 and pLOC overexpression of Mcl‐1 were developed in the THP‐1 cell line (Figure 4C and D). Consistent with our previous studies and our hypothesis, Mcl‐1 knockdown greatly enhanced ABT‐199‐induced apoptosis (Figure 4C). It also enhanced apoptosis induced by ABT‐199 and KPT‐330 combination treatment. On the other hand, Mcl‐1 overexpression partially rescued the cells from drug treatment (Figure 4D). The incomplete rescue may be due to the Mcl‐1 overexpression not being high enough and/or potential Mcl‐1‐independent mechanisms through which KPT‐330 acts.

To begin to understand the molecular mechanism by which KPT‐330 down‐regulates Mcl‐1, real‐time RT‐PCR was performed in THP‐1 and OCI‐AML3 cell lines post KPT‐330 and ABT‐199 treatment for 24 hours. Interestingly, Mcl‐1 transcript levels were not decreased by ABT‐199 or KPT‐330 treatment in both cell lines (Figure 4E and F), indicating a post‐transcriptional mechanism. To test this possibility, the two cell lines were treated with or without KPT‐330 for 12 hours, then washed and cultured in the presence of the protein translation inhibitor cycloheximide for up to 120 minutes. KPT‐330 treatment resulted in significantly shorter Mcl‐1 half‐life in both THP‐1 and OCI‐AML3 cells (Figure 4G and H), demonstrating that KPT‐330 decreases Mcl‐1 protein stability. In summary, Mcl‐1 is post‐transcriptionally modulated by KPT‐330 treatment alone and in combination with ABT‐199, and this modulation of Mcl‐1 plays an important role in apoptosis in response to KPT‐330 and ABT‐199 treatment.

### 3.3 KPT‐330 synergizes with ABT‐199 in primary AML patient samples

**Figure 5:**
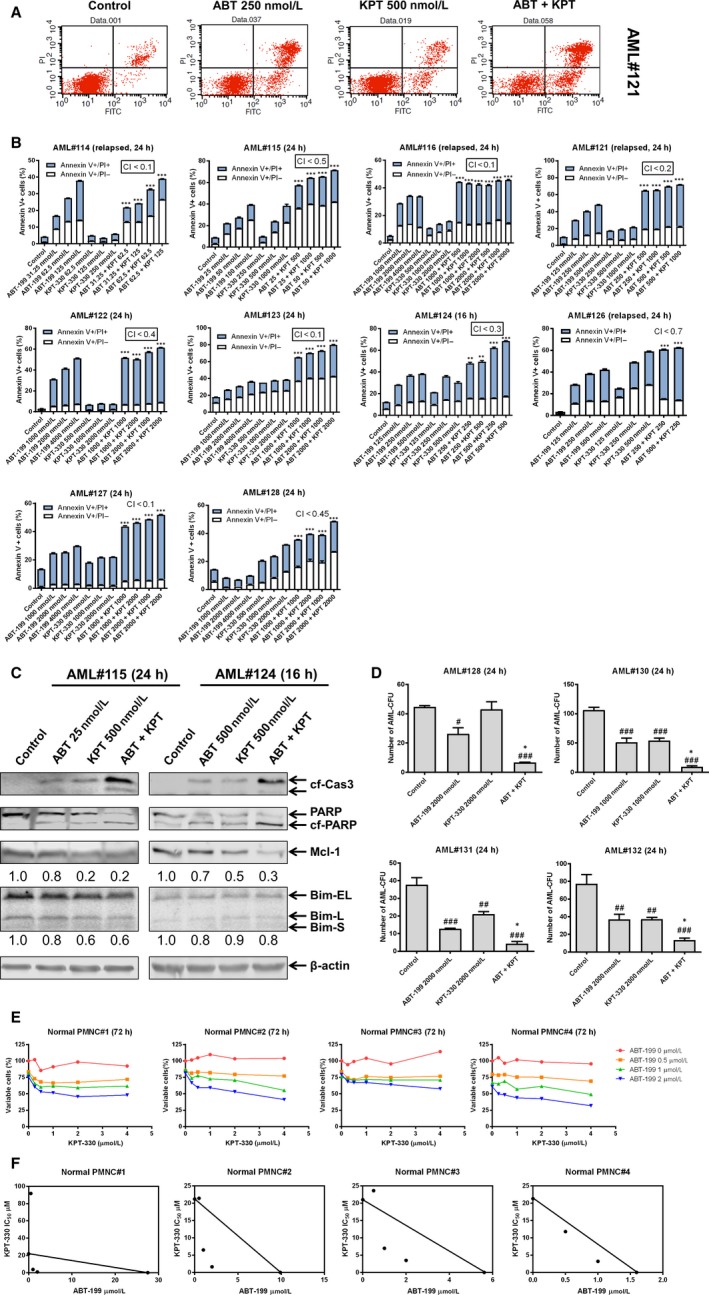
KPT‐330 synergizes with ABT‐199 to induce apoptosis and cooperatively reduce colony formation in primary AML patient samples. (A and B) Primary AML patient samples were treated with ABT‐199 and/or KPT‐330 for 24 hours and then subjected to Annexin V‐FITC/PI staining and flow cytometry analyses. Representative dot plots are shown for primary patient AML#121 (A); ***P < 0.001. Combination index (CI) values were calculated using CompuSyn software. (C) Primary AML patient samples were treated with ABT‐199 and/or KPT‐330 for 24 hours. Whole cell lysates were subjected to Western blotting and probed with the indicated antibodies. (D) Primary AML patient samples were treated with ABT‐199 and/or KPT‐330 for 24 hours and then plated in methylcellulose (n = 3). Colonies were counted manually. ^#^ *P* < 0.05 vs control; ^##^ *P* < 0.01 vs control; ^###^ *P* < 0.001 vs control; *P < 0.05 vs single drug treatment; **P < 0.01 vs single drug treatment. (E and F) Normal PMNCs from healthy donors were treated with variable concentrations of KPT‐330 alone and in combination with ABT‐199 for 72 hours. MTT assays were used to determine viable cells. Results are graphed as percentage of viable cells compared to vehicle control (E). Standard isobologram analysis was performed. The IC_50_ values of each drug are plotted along the axes. The solid line connecting the single drug IC_50_s indicates an additive effect; points falling above the line indicate antagonism, while those below the line indicate synergism

To determine if KPT‐330 can synergize with ABT‐199 to induce apoptosis in primary AML patient samples ex vivo, we tested various concentrations of ABT‐199 and KPT‐330, alone and in combination, in ten primary AML patient samples. At 24 hours, synergy was observed between the two drugs for all samples (Figure 5A and B). Cleavage of PARP and caspase 3 was enhanced in the combination treatment when compared to ABT‐199 or KPT‐330 alone (Figure 5C). Similar to AML cell lines, KPT‐330 alone and in combination with ABT‐199 decreased Mcl‐1 levels. To determine the effect of KPT‐330 and ABT‐199 on AML progenitor cells, we performed colony formation assays on primary AML patient samples post drug treatment. As shown in Figure 5D, individual drug treatment significantly decreased AML‐CFUs, except KPT‐330 treatment in AML#128, while the combination treatment significantly enhanced inhibition of colony formation when compared to single drug treatment (Figure 5D), demonstrating that KPT‐330 and ABT‐199 cooperate in killing AML progenitor cells in vitro. Next, the effects of ABT‐199 and KPT‐330 on normal hematopoietic cells were tested by MTT assays in four normal PMNC samples. KPT‐330 treatment had little to no effect on viable cells, while ABT‐99 treatment reduced the percentage of viable cells compared to no treatment control (Figure 5E). The combination treatment showed additive to synergistic effect on the normal PMNCs, suggesting that toxicity could be a concern (Figure 5F). In summary, ABT‐199 and KPT‐330 can synergize to induce apoptosis in bulk AML cells in vitro and significantly reduce AML progenitor cells ex vivo.

## 4 DISCUSSION

ABT‐199 has demonstrated clinical activity, though as a monotherapy relapse occurred in a mere 2.5 months and in combination with decitabine or azacitidine there was no significant improvement in overall survival rate compared to decitabine or azacitidine alone.^18^, ^19^, ^20^ Therefore, new combination approaches for ABT‐199 are necessary to improve the long‐term survival of AML patients. In this study, we show that KPT‐330 is able to overcome ABT‐199 resistance by decreasing Mcl‐1 stability and enhancing AML cell death. Lapalombella reported that inhibition of XPO1 in CLL cells decreased Mcl‐1 mRNA.^29^ In contrast, we did not detect a decrease of Mcl‐1 mRNA following KPT‐330 treatment. However, we did identify that KPT‐330 decreased Mcl‐1 protein stability (Figure 4). While down‐regulation of Mcl‐1 played a role, it was detected after initiation of apoptosis. However, immunoprecipitation of Bim indicates that KPT‐330 prevents increased binding of Bim to Mcl‐1 in response to ABT‐199 treatment at 8 hours (Figure 3C). Surprisingly, KPT‐330 treatment resulted in increased binding of Bcl‐2 to Bim in AML cell lines (Figure 4B). These results indicate that down‐regulation of Mcl‐1 by KPT‐330 results in release of Bim from Mcl‐1; however, the released Bim is sequestered by Bcl‐2 preventing it from inducing apoptosis, representing a mechanism of apoptosis resistance to KPT‐330. This is analogous to the apoptosis resistance to ABT‐199 mediated by Mcl‐1 in AML cells.^21^ Interestingly, the combination of KPT‐330 and ABT‐199 abolished both mechanisms of apoptosis resistance, demonstrating that the two agents reciprocally overcome resistance to single agent treatment. Though how treatment with KPT‐330 prevents Mcl‐1 from binding to Bim remains unknown, it is possible that KPT‐330 treatment affects modification of Mcl‐1 and/or Bim, resulting in decreased binding between the two proteins. Further studies are necessary to determine how this occurs, but are beyond the scope of this study.

Additional questions remain regarding the combination treatment's mechanism of action and the mechanisms of ABT‐199 resistance. While the combination of KPT‐330 and ABT‐199 synergistically induced apoptosis in all ten primary AML patient samples tested, there was considerable variation in the response. The mechanism of heterogeneity remains unclear. Both ABT‐199 and KPT‐330 modulate Mcl‐1 levels through protein stability (Figure 4 and ref. 21). Mcl‐1 stability is known to be affected by phosphorylation sites (e.g. S159 and T163) as well as binding to Bim, which can be disrupted by post‐translational modifications.^49^ These sites can be regulated by a host of other signalling molecules (e.g. GSK3β, p38, JNK, ERK) whose roles in this combination have yet to be determined. In addition, E3 ligases such as Mule, β‐TrCP, FBW7, Trim17, and SCFβ, and deubiquitinases USP9X and USP28 may also be altered to affect Mcl‐1 stability.^50^ Bim binds to the same BH3 binding pocket on Mcl‐1 as these E3 ligases and its disrupted interaction with Mcl‐1 may increase Mcl‐1 interaction with these E3 ligases and alter Mcl‐1 stability.

In summary, KPT‐330 synergizes with ABT‐199 to induce apoptosis in AML cell lines and primary patient samples at clinically relevant concentrations.^12^, ^32^, ^51^, ^52^ Both Bcl‐2 and XPO1 are up‐regulated in LSCs and their inhibition by ABT‐199 and KPT‐330, respectively, can selectively target LSCs.^2^, ^35^ Importantly, we show that the combination cooperatively inhibits AML primary patient sample leukaemic progenitor cells ex vivo, suggesting that the combination of ABT‐199 and KPT‐330 may show activity against LSCs. However, the combination does appear to synergize in normal PMNC cells ex vivo, suggesting that toxicity could be a concern. Further in vitro mechanistic studies and in vivo testing in AML mouse models is warranted to determine efficacy against LSCs as well as tolerability.

## CONFLICT OF INTEREST

The authors declare no competing financial interests.

## AUTHOR CONTRIBUTION

YG designed the study; DAL, YS, SL performed the experiments; YG, JWT, YW, HL, HE, DAL, YS, and SL analysed and interpreted the data; YG, HE, DAL, JWT, HL, and YW wrote the manuscript. All authors read and approved the final manuscript.
